# Predominance of Distinct *Listeria Innocua* and *Listeria* *Monocytogenes* in Recurrent Contamination Events at Dairy Processing Facilities

**DOI:** 10.3390/microorganisms8020234

**Published:** 2020-02-10

**Authors:** Irene Kaszoni-Rückerl, Azra Mustedanagic, Sonja Muri-Klinger, Katharina Brugger, Karl-Heinz Wagner, Martin Wagner, Beatrix Stessl

**Affiliations:** 1 Unit of Food Microbiology, Institute of Food Safety, Food Technology and Veterinary Public Health, Department of Farm Animal and Public Health in Veterinary Medicine Department of Veterinary Public Health and Food Science, University of Veterinary Medicine Vienna, Veterinärplatz 1, 1210 Vienna, Austria; irene.rueckerl@gmx.at (I.K.-R.); sonja.klinger@vetmeduni.ac.at (S.M.-K.); martin.wagner@vetmeduni.ac.at (M.W.); 2 Department of Nutritional Sciences, Faculty of Life Sciences, University of Vienna, Althanstraße 14, 1090 Vienna, Austria; karl-heinz.wagner@univie.ac.at; 3 Austrian Competence Center for Feed and Food Quality, Safety and Innovation (FFOQSI), Technopark C, 3430 Tulln, Austria; azra.mustedanagic@ffoqsi.at; 4 Unit of Veterinary Public Health and Epidemiology, Institute of Food Safety, Food Technology and Veterinary Public Health, Department of Farm Animal and Public Health in Veterinary Medicine Department of Veterinary Public Health and Food Science, University of Veterinary Medicine Vienna, Veterinärplatz 1, 1210 Vienna, Austria; Katharina.Brugger@vetmeduni.ac.at

**Keywords:** *Listeria monocytogenes*, *Listeria innocua*, dairy processing, subtyping, persistence

## Abstract

The genus *Listeria* now comprises up to now 21 recognized species and six subspecies, with *L. monocytogenes* and *L. innocua* as the most prevalent sensu stricto associated species. Reports focusing on the challenges in *Listeria* detection and confirmation are available, especially from food-associated environmental samples. *L. innocua* is more prevalent in the food processing environment (FPE) than *L. monocytogenes* and has been shown to have a growth advantage in selective enrichment and agar media. Until now, the adaptive nature of *L. innocua* in FPEs has not been fully elucidated and potential persistence in the FPE has not been observed. Therefore, the aim of this study is to characterize *L. innocua* (*n* = 139) and *L. monocytogenes* (*n* = 81) isolated from FPEs and cheese products collected at five dairy processing facilities (A–E) at geno- and phenotypic levels. Biochemical profiling was conducted for all *L. monocytogenes* and the majority of *L. innocua* (*n* = 124) isolates and included a rhamnose positive reaction. *L. monocytogenes* isolates were most frequently confirmed as PCR-serogroups 1/2a, 3a (95%). Pulsed-field gel electrophoresis (PFGE)-typing, applying the restriction enzymes *AscI*, revealed 33 distinct *Listeria* PFGE profiles with a Simpson’s Index of Diversity of 0.75. Multi-locus sequence typing (MLST) resulted in 27 STs with seven new *L. innocua* local STs (ST1595 to ST1601). *L. innocua* ST1597 and ST603 and *L. monocytogenes* ST121 and ST14 were the most abundant genotypes in dairy processing facilities A–E over time. Either SSI-1 (ST14) or SSI-2 (ST121, all *L. innocua*) were present in successfully FPE-adapted strains. We identified housekeeping genes common in *Listeria* isolates and *L. monocytogenes* genetic lineage III. Wherever there are long-term contamination events of *L. monocytogenes* and other *Listeria* species, subtyping methods are helpful tools to identify niches of high risk.

## 1. Introduction

The genus *Listeria,* assigned to the phylum Firmicutes, comprises Gram-positive facultative anaerobe bacteria that are found ubiquitously in environments such as soil, water, or plant material [1,2]. The genus comprises up to 21 recognized species and six subspecies, with *L. monocytogenes* and *L. innocua* as the most prevalent sensu stricto associated species [3] (http://www.bacterio.net/listeria.html). *L. monocytogenes* has been implicated in human listeriosis outbreaks, most often associated with ready-to-eat (RTE) food products (https://www.cdc.gov/listeria/outbreaks/index.html) [4,5]. Food is most commonly contaminated by *L. monocytogenes* from niches in the food processing environment (FPE) [6]. As an environmental saprophyte, *L. monocytogenes* is highly adapted to harsh conditions, such as those associated with osmotic and cold stress, low pH, desiccation, and competitive microflora [7]. The adaptive strategies of *L. monocytogenes* to ecological niches are clearly divergent. Genetic lineage I (serovar 1/2b and 4b) has a tropism to human and animal host tissues and cell types, whereas genetic lineage II (serovar 1/2a and 1/2c) is more adapted to environmental conditions [8,9].

The ecological co-habitation, genomic synteny, and phenotypic similarities of *L. monocytogenes* and *L. innocua* qualify the latter as surrogate for *L. monocytogenes* behavior prediction in FPEs [10]. Nevertheless, the *prfA*-virulence gene cluster that was present in the common ancestor of *Listeria* species was lost in two separate recombination events with *L. innocua* and *L. welshimeri* [11]. In fact, *L. innocua* comprises two major subgroups A and B, with one atypical subgroup D (e.g., hemolytic, LIPI regions and *inlJ* positive) serving as a link between *L. monocytogenes* and *L. innocua* in the evolutionary chain [12]. Internalin genes (*lin0354, lin0661, lin1204, and lin2539*) have been suggested as potential genetic markers for *L. innocua* subgroups A-D [13]. Hemolytic *L. innocua* strains have now been characterized by whole genome sequencing (WGS) and virulence testing. The strains harbor a pathogenic island, LIPI-1, and internalin *inlA,* required for mammalian cell invasion. These findings will challenge risk management in the food chain, as *L. innocua* was often assessed in the past as non-hazardious [12]. Currently, *L. innocua* genome sequences are still limited [14,15]. Eleven *L. innocua* genome assemblies are currently available from the National Center for Biotechnology Information (NCBI) (https://www.ncbi.nlm.nih.gov/genome/genomes/1024?).

The loss of full virulence in *L. monocytogenes* lineage II strains (e.g., sequence type (ST) 9, ST121), due to point mutations in *prfA, inlA, inlB,* and *plcA,* indicates the formation of monophyletic groups present in FPEs [16]. The latter strains were mentioned, amongst others (e.g., ST5, ST7, ST8), in past long-term FPE contamination scenarios, which were then referred to as in-house clones or persisters [17,18,19,20]. Until now, the adaptive nature of *L. innocua* in FPEs has not been fully characterized and potential persistence has not been observed. Accordingly, the aim of this study is to characterize potential *L. innocua* persistent isolates originating from FPEs and cheese products on geno- and phenotypic levels and to compare them to *L. monocytogenes* strains from the same habitat.

## 2. Materials and Methods

### 2.1. Isolate Selection and Listeria Species Confirmation

In total, 220 *Listeria* isolates (139 *L. innocua* and 81 *L. monocytogenes*) originating from different cheese types, product-associated liquids (PAL; smear, brine), product-associated samples (culture, enrichment), raw material (RM), food contact surfaces (FCS), non-food contact surfaces (NFCS), and environmental liquid samples (EL; floor and drain water) were included in this study. The isolates were collected during a *Listeria* monitoring program between 1987 and 2010 from five dairy producers (A–E) [21,22]. The selection criteria for this study were as following: (1) producer with a history of frequent *L. innocua* or *L. monocytogenes* isolation, and (2) *L. monocytogenes* previously identified as potentially persistent in the FPE. Producer A was mainly involved with ripening cheese and packaging grated and sliced semi-hard cheeses. Producers B and C manufactured a broad range of white dairy and fresh products and a product line of semi-hard and hard cheeses. Producer D manufactured a range of semi-hard and red smear cheeses and producer E manufactured acid curd cheeses (Table 1). The *L. innocua* and *L. monocytogenes* isolates were stored at −80 °C at the *Listeria* collection located at the Unit of Food Microbiology, University of Veterinary Medicine Vienna, Austria. *L. innocua* and *L. monocytogenes* isolates were re-cultivated on tryptic soy agar (TSA; Merck KgA, Darmstadt, Germany) at 37 °C overnight. DNA isolation followed a rapid protocol using Chelex 100-Resin (Bio-Rad Laboratories Inc., Hercules, CA, USA), as published by Walsh et al. [23]. For *Listeria* species differentiation, a multiplex PCR targeting the invasion-associated protein (*iap*) gene was performed [24]. *L. monocytogenes* isolates were subtyped by serogroup PCR, targeting the *Listeria* spp. specific *prs*, and *lmo0737*, *lmo1118*, *ORF2819*, and *ORF2110* [25]. Biochemical profiling was performed for each *Listeria* isolate by applying the API-*Listeria* identification system (Biomérieux, Marcy l’Etoile, France).

To evaluate a possible association between the categorical variables, i.e., *Listeria* species, rhamnose (yes/no), and sporadic/persistence, the association coefficient (Cramer’s V) was calculated (Microsoft Excel 2010, Microsoft Corp., Redmond, WA, USA).

### 2.2. Molecular Epidemiological Analysis

DNA macrorestriction digest, applying *Asc*I and *Apa*I of 220 *Listeria* isolates (139 *L. innocua* and 81 *L. monocytogenes*), was performed according to the latest CDC PulseNet International PFGE protocol (https://www.cdc.gov/pulsenet/pdf/listeria-pfge-protocol-508c.pdf). In brief, the cell suspension was standardized to an optical density of 1.0 in sterile TE buffer (10 mM Tris, 1 mM EDTA, pH 8; Sigma-Aldrich Corp, St. Louis, MO, USA) and lysed by adding 20 mg/mL lysozyme (Sigma-Aldrich Corp) to an incubation step at 55 °C for 30 min. After incubation, 20 mg/mL proteinase K (Roche Diagnostics GmbH, Mannheim, Germany) was added to the cell suspension followed by 1% SeaKem Gold agarose (1:1; each 400 µL; Lonza Group, Basel, Switzerland). The suspension was poured into plug molds and the solidified plugs were lysed overnight in 5 mL of cell lysis buffer (50 mM Tris, 50 mM EDTA, pH 8, 1% Sarcosyl + 0.1 mg/mL proteinase K; Sigma-Aldrich Corp). Subsequently, the plugs were washed with sterile water (twice; Mayrhofer Pharmazeutika, Leonding, Austria) and TE buffer (three times). The *Listeria* DNA macrorestriction digest was performed by applying 50 U each of *AscI* and *ApaI* at 37 °C and 30 °C for 4 h (Thermo Fisher Scientific Inc., Waltham, MA, USA). The universal standard *Salmonella* ser. Braenderup H9812 was digested with 50 U *Xba*I (Thermo Fisher Scientific Inc.) at 37 °C for 4 h (https://www.cdc.gov/pulsenet/pdf/ecoli-shigella-salmonella-pfge-protocol-508c.pdf). The restriction digested plugs were loaded into 1% SeaKem Gold Agarose gel in 0.5 × Tris borate EDTA buffer (45 mM Tris, 45 mM borate, 1 mM EDTA; Sigma-Aldrich Corp) and electrophorezed for 22.5 h at 6 V/cm with a linear ramping factor and pulse times from 4.0 to 40.0 s at 14 °C and an included angle of 120° (CHEF DR III system; Bio-Rad Laboratories Inc., Hercules, CA, USA).

The gel was stained with ethidium bromide (Sigma-Aldrich Corp) and digitally photographed with Gel Doc 2000 (Bio-Rad Laboratories, Inc.). The TIFF images were normalized with BioNumerics 6.6 software package (Applied Math NV, Sint-Martens-Latem, Belgium) to the universal standard *Salmonella* ser. Braenderup H9812. Pattern clustering utilized the unweighted pair group method with arithmetic mean (UPGMA) and the dice correlation coefficient with a position tolerance of 1.5%. PFGE types were considered identical when the patterns were indistinguishable. The Simpson’s Index of diversity was calculated with the online tool of Comparing Partitions (http://www.comparingpartitions.info/).

The *L. monocytogenes* multi-locus sequence typing (MLST) scheme included the following seven housekeeping genes: ABC transporter (*acbZ, lmo*2752), beta glucosidase (*bglA, lmo*0319), catalase (*cat, lmo*2785), succinyl diaminopimelate desuccinylase (*dapE, lmo*0265), D-amino acid aminotransferase (*dat, lmo*1617), L-lactate dehydrogenase (*ldh, lmo*0210), and histidine kinase (*lhkA, lmo*1508). Protocols for target-specific primers and PCR conditions are provided at https://bigsdb.web.pasteur.fr/listeria/primers_used.html. Target-specific PCR products were sequenced with universal sequencing primers (*oF*: GTT TTC CCA GTC ACG ACG TTG TA; *oR*: TTG TGA GCG GAT AAC AAT TTC; LGC Genomics, Berlin, Germany) and allele-specific sequences were submitted to the Institute Pasteur sequence and profile database (https://bigsdb.pasteur.fr/cgi-bin/bigsdb/bigsdb.pl?db=pubmlst_listeria_seqdef). The sequence types (ST) were determined by the combination of the seven housekeeping loci. The STs were compared to the Institute Pasteur isolate database (https://bigsdb.pasteur.fr/cgi-bin/bigsdb/bigsdb.pl?db=pubmlst_listeria_isolates_public) to estimate their global presence and potential niche attribution.

### 2.3. Screening for Stress Survival Islets (SSI-1 and SSI-2)

*L. innocua* and *L. monocytogenes* isolates were screened for the presence of SSI-1^+^ (9.7 kbp fragment), SSI-1^-^(F2365_0481 homologous gene; 1.1 kbp fragment) and SSI-2 (2.2 kbp fragment). PCR primers targeting the *L. monocytogenes* flanking genes *lmo0443* and *lmo0449* were used according to Ryan et al. [26]. The homologous genes related to *L. innocua* *lin0464* and *lin0465* (2.2 kbp fragment) were investigated according to Hein et al. [27]. PCR reactions contained 0.2 μM each primer, 2 mM MgCl_2_, 1 mM deoxynucleoside triphosphates (dNTPs; Thermo Fisher Scientific), 1 U Platinum *Taq* DNA polymerase (Thermo Fisher Scientific), 10× PCR buffer, diethyl pyrocarbonate (DEPC)-treated water (Thermo Fisher Scientific), and 1 μL DNA template in a final volume of 25 μL.

The PCR reaction for the detection of SSI-1^+^ (9.7 kbp) and SSI-1^+^ (1.1 kbp) differed from the latter mix by the following components: 2.5 U long range DNA polymerase and 2 μL DNA template in a final volume of 25 μL. The gel electrophoresis of PCR-reactions was determined in a 1.5% agarose gel containing 0.5 × Tris–borate–EDTA (TBE) buffer and 3.5 μL peqGREEN DNA gel stain (VWR International, Radnor, PA, USA). The DNA standard Thermo Scientific™ GeneRuler™ 100 bp and 1 kb plus (Thermo Fisher Scientific Inc.) were applied for fragment length comparison.

### 2.4. L. Monocytogenes and L. Innocua Minimum Inhibitory Concentration (MIC) towards Biocides

The minimum inhibitory concentrations (MIC) of five disinfectant compounds (peracetic acid, benzalkonium chloride, sodium hypochlorite, hydrogen peroxide, and isopropanol; all supplied by Sigma AldrichCorp) were determined for 10 recurrent *L. monocytogenes* and *L. innocua* genotypes: M1[E] (ST59, 1/2b); M5[B]=M5[D]=M5[E] (ST121, 1/2a); M10[C] (ST155, 1/2a); M11[A]=M7[D] (ST14, 1/2a); IN1[E] (ST1595); IN2[E] (ST637); IN3[E] (ST1601); IN4[C]=IN4 [E] (ST603); IN5[A]=IN5[C]=IN5[D]=IN5[E] (ST1597); IN7[C] (ST1085).

The disinfectant components were tested at concentration ranges of 31.3–1000 mg/L for peracetic acid and hydrogen peroxide, 0.5–1000 mg/L for benzalkonium chloride and 125–10,000 mg/L for sodium hypochlorite. An agar dilution method was performed in duplicates to determine the minimal inhibitory concentration (MIC) of the disinfectants against *L. innocua* and *L. monocytogenes* strains. As previously described, 5 µL of bacterial culture was spotted onto Mueller–Hinton agar (Oxoid, Basingstoke, UK) containing the disinfectants to be tested [28,29]. Plates were incubated at 37 °C for 24 to 48 h. Following incubation, the lowest disinfectant concentration that showed no bacterial growth was recorded as MIC. Mean MIC values were calculated using Excel (Microsoft Corporation, Redmond, WA, USA).

## 3. Results

### 3.1. Isolate Characteristics

The isolate set comprised 139 *L. innocua* and 81 *L. monocytogenes* PCR-confirmed isolates targeting the *iap* (invasion associated protein p60) gene [24]. *Listeria* spp. originated from the following Austrian cheese processing facilities: A (*L. innocua/L. monocytogenes*: (*n* = 9/13), B (*n* = 0/47), C (*n* = 34/3), D (*n* = 72/9), and E (*n* = 24/9). The *Listeria* isolate collection was established over a monitoring period of 23 years (1987–2010). *Listeria* spp. were isolated from cheese samples (24 *L. innocua* and 3 *L. monocytogenes*), product associated samples (PA, PAL; 100 *L. innocua* and 64 *L. monocytogenes),* production environment (FCS, NFCS, environmental liquids (EL); 14 *L. innocua* and 14 *L. monocytogenes),* and one *L. innocua* isolate from raw milk (RM) (Table 1).

The biochemical profiling resulted for all *L. monocytogenes* isolates in the typical naphthylamidase (DIM; Differentiation/*Innocua*/*Monocytogenes*) negative and rhamnose positive profile (API profile 6510). *L. innocua* isolates exhibited three different biochemical profiles: API profile 7510 with a DIM and rhamnose positive reaction (*n* = 124), API profile 7110 with a DIM positive and rhamnose negative reaction (*n* = 13), and API profile 7531 with a DIM, D-ribose, and D-tagatose positive reaction (*n* = 2) (Table 1).

*L. monocytogenes* isolates (*n* = 81) were most frequently confirmed as PCR-serogroups 1/2a, 3a (*n* = 76; 95%), followed by 1/2b, 3b (*n* = 3; 3.75%), and 4b, 4d, 4e (*n* = 2; 1.25%).

The PFGE-typing, applying the restriction enzyme *AscI,* revealed 33 distinct *Listeria* PFGE profiles with a Simpson’s Index of Diversity of 0.75. Thereof, 11 and 22 *Asc*I were specific for *L. monocytogenes* and *L. innocua,* respectively (Simpson´s Index 0.519 and 0.533). The *Apa*I macrorestriction digest resulted in fewer PFGE profiles for *L. innocua* (*n* = 20). *L. innocua* PFGE profiles IN5[A]=IN5[C]=IN5[D]=IN5[E] and IN4[C]=IN4[E] were non-typable by *Apa*I.

**Figure 1:**
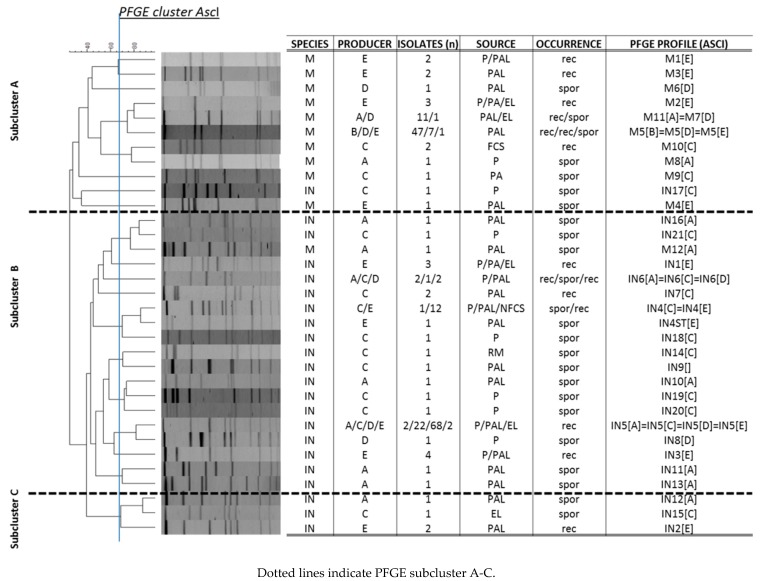
PFGE cluster analysis (*Asc*I) of *L. innocua* (*n* = 139) and *L. monocytogenes* (*n* = 81) isolates included in this study.

PFGE-types with identical *Asc*I profiles assigned to dairy producers A-E and isolated during two or more sampling events were classified as recurrent, whereas *Listeria* isolates with unique *Asc*I profiles and isolated once were defined as sporadic genotypes. *Listeria* spp. genotypes recurrently isolated over a period of six or more months were defined as persistent. Generally, *L. monocytogenes* clustered together in a distinct subcluster A and could be clearly distinguished at a similarity level of 25% from *L. innocua* subcluster B and C (similarity level 40%) (Table 1, Figure 1).

The MLST typing resulted in 27 STs (Simpson´s Index of Diversity of 0.742). In total, 9 and 3 clonal complexes (CCs) and 1 and 14 singletons were identified among *L. monocytogenes* and *L. inncoua* isolates, respectively. The discriminatory power of MLST analysis was comparable to PFGE-typing for *L. monocytogenes* (10 STs; Simpson´s Index 0.469). ST121 could be differentiated by applying PFGE typing into two *L. monocytogenes* distinct fingerprints. MLST analysis for *L. innocua* isolates was less discriminative in comparison to PFGE (*Asc*I), resulting in 17 STs (Simpson´s Index 0.531).

**Figure 2:**
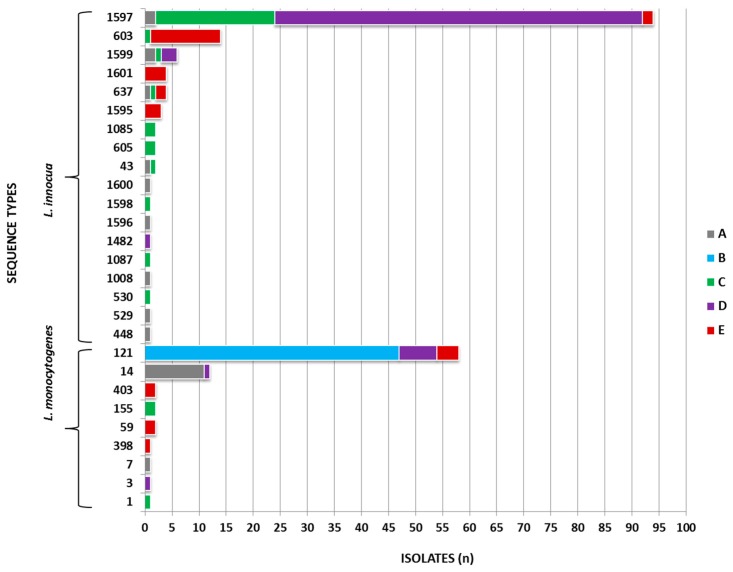
Comparison of the *L. innocua* and *L. monocytogenes* ST*s* identified in this study to the Institute Pasteur MLST isolate database (https://bigsdb.pasteur.fr/listeria/listeria.html) was performed to identify highly abundant housekeeping genes, CC and STs (Table S1). Interestingly, some housekeeping genes were not specific for *L. monocytogenes* genetic lineage III or *L. innocua*.

Seven new *L. innocua* STs (ST1595 to ST1601) were defined by submitting the sequences to the Institute Pasteur MLST database (https://bigsdb.web.pasteur.fr/listeria/listeria.html; Table 1). The ST attribution to dairy processing facilities A–E is depicted in Figure 2.

The stress survival islet (SSI-1) inserted into intergenic region *lmo0443* to *lmo0449* in *L. monocytogenes* was present in ST3 (genetic lineage I; PCR serogroups 1/2b, 3b), ST7, ST14, ST155, and ST403 (genetic lineage II; PCR serogroup 1/2a, 3a). The *L. monocytogenes* homologous gene to *F2365_0481* (1.1 kbp) was present in genetic lineage I isolates assigned to ST1 (serogroup 4b, 4d, 4e) and ST59 (1/2b, 3b), ST398 (genetic lineage II (PCR-serogroup 1/2a, 3a), and ST529 (genetic lineage III, PCR-serogroup 4b, 4d, 4e). *L. monocytogenes* ST121 and all *L. innocua i*solates harbored the SSI-2 (2.2 kbp fragment; Table 1).

### 3.2. Molecular Epidemiological Interpretation

The majority of *L. innocua* and *L. monocytogenes* PFGE types were isolated once (*n* = 20/33; 60.61%), but certain genotypes were recurrently isolated from process associated samples and cheese for a short period of time (*n* = 6/33; 18.18%). These genotypes were present in PAL (smear) and environmental samples and after contamination events in cheese for between one and six months and were successfully eliminated. Other *Listeria* spp. PFGE types were persistent in the dairy processing environment for a long period and somehow adapted to niches (smear, brine, drain water). The latter *L. monocytogenes* and *L. innocua* PFGE types cross-contaminated the surface of cheeses (e.g., hard cheese). Almost all of the persistent *L. innocua* and *L. monocytogenes* isolates were rhamnose positive (API profiles 7510 and 6510), except for one genotype with a rhamnose negative profile (IN7[C], ST1085). Almost all of the persistent *L. innocua* and *L. monocytogenes* isolates were rhamnose positive (API profiles 7510 and 6510) except for one genotype with a rhamnose negative profile (IN7[C], ST1085). The association coefficient Cramer’s V showed a weak association between *L. innocua*, *L. monocytogenes*, rhamnose positive, rhamnose negative sporadic and persistent occurrences, although the result “for persistence and rhamnose positive” was highly significant (*p* = 0.0065; Cramer’s V rV = WERT, *p* < 0.01).

The most abundant *L. monocytogenes* genotypes related to persistence in cheese processing associated samples and environments were PFGE profiles M5[B]=M5[D]=M5[E] (ST121, 1/2a, 3a) and M11[A]=M7[D] (ST14, 1/2a, 3a), which were present for 7 and 11 years at dairy processing facility B and A in smear and drain water, respectively. The latter profiles were also isolated once and after four months at producers D and E. PFGE profiles M10[C] (ST155, 1/2a, 3a), M3[E] (ST403, 1/2a, 3a) were present at producers C and E for one year and seven months. M1 (ST59, 1/2b, 3b) and M2 (ST121, 1/2a, 3a) were recurrently isolated during a shorter timeframe (one and two months), both at producer E. Other sporadically isolated *L. monocytogenes* genotypes (ST1, ST3, ST7, and ST398) were isolated once during the monitoring period.

The most common *L. innocua* genotypes were IN5[A]=IN5[C]=IN5[D]=IN5[E] (newly identified ST1597), IN4[C]=IN4[E] (ST603), and IN7[C] (ST1085), which were repeatedly isolated for a year up to 6.8 years in the same dairy processing environment (floor water) and product-associated liquids (smear, brine). IN1[E] (newly identified ST1595) and IN 3[E] (newly identified ST1601) were recurrently isolated during a six-month period at processing facility E.

IN6[A]=IN6[C]=IN6[D] was present during a short contamination event (four and five months) at processing facilities A and D. The *L. monocytogenes* and *L. innocua* PFGE clusters were heterogeneous (25% similarity). Interestingly, the persistence-related genotypes *L. monocytogenes* ST14 (M11[A]=M7[D]), ST155 (M10[C]), and ST121 (M5[B]=M5[D]=M5[E]) clustered together in subcluster A at a 75% and 80% similarity level. *L. innocua* ST43 (IN16[A], IN21[C]), ST603 (IN4ST[E], IN4[C]=IN4[E]), and ST637 (IN12[A], IN15[C]) related PFGE profiles clustered in subcluster B and C at a similarity level ≥80%.

*L. innocua* ST1597, ST603 and *L. monocytogenes* ST121 and ST14 were the most abundant genotypes in dairy producing facilities A–E over time (*n* = 178/220 isolates). The highest genotype diversity was identified in dairy producing facilities A, C, and E (*n* = 11, 12, and 9 different genotypes; Figure 2).

The following housekeeping genes were common in *Listeria* isolates included in this study and *L. monocytogenes* genetic lineage III: ST529, ST1595 (*abcZ* 25, *bglA* 73), ST529, ST1597 and ST1599 (*dapE* 96), ST529, ST530, ST448, ST1482, ST605, and ST1087 (*dat* 45) (Table 1). Furthermore, the housekeeping gene *abcz* 40 in *L. innocua* ST1600 was identified in the Institute Pasteur MLST database to be more related to *L. monocytogenes* genetic lineage III (ST267). Further details concerning the prevalence of housekeeping genes present in the MLST database are provided in Table S1.

### 3.3. Susceptibility to Biocides

The MIC towards biocides was determined for four recurrent *L. monocytogenes* and six *L. innocua* genotypes. All test strains except *L. innocua* genotype IN5[A]=IN5[C]=IN5[D]=IN5[E] (ST1597) and IN3[E] (ST1601), both 141 mg/L, were adapted to higher concentrations of peracetic acid (250 mg/L; 1.7 fold higher). M5[B]=M5[D]=M5[E] (ST121, 1/2a), IN5[A]=IN5[C]=IN5[D]=IN5[E] (ST1597), and IN2[E] (ST637) were better adapted to benzalkonium chloride (1.3- and 2-fold higher; mean MIC 15.6 mg/L in contrast to 11.7 and 7.8 mg/L). M5[B]=M5[D]=M5[E] (ST121, 1/2a) and M1[E] (ST59, 1/2b) were slightly better adapted to hydrogen peroxide (1.5-fold higher; 188 mg/L in contrast to 125 mg/L). IN1[E] (ST1595) and IN2[E] (ST637) were better adapted to sodium hypochlorite (2.7–5.7-fold higher; 10,000 mg in contrast to 1750–3750 mg/L).

## 4. Discussion

Dairy and cheese processing environments are frequently colonized by *Listeria* spp., including pathogenic *L. monocytogenes*. Even newly established dairy processing facilities become colonized after a short period of time [29,30].

Generally, prevalence and concentrations of *L. monocytogenes* in cheeses and cheese processing environments are low. Its growth is supported by the presence of fresh, ripened, veined, and smear cheeses (0.8%–5.1% prevalence in cheese lots). Brined cheeses are most often contaminated by *L. monocytogenes* (11.8%), according to a meta-analysis-based literature review [31]. This suggests that product-associated liquids (smear, brine) contribute to *L. monocytogenes* contamination of cheese lots [21,32]. In our study, smear and brine samples were indeed most often associated with *L. monocytogenes* and *L. innocua* surface contamination and supported the persistence of certain genotypes (ST121, ST14, ST603, and ST1597) in the dairy environment.

Floor drains are further niches for efficient *Listeria* spp. colonization of the FPE and hot-spots for cross and recontamination events [33,34]. These niches were also identified in our study. To a certain extent, drain waters harbored recurrent and persistent *Listeria* spp. (ST14, ST637, ST1595, ST1597; Table 1). Despite having a cleaning potential, the introduction of high-pressure water from hoses into a contaminated drain can cause the airborne spread of *Listeria* and further contribute to the successful establishment of persistent *Listeria* spp. strains in a facility [35].

As reports about dairy processing environment contamination scenarios in the literature are sparse, our goal was to identify any potential long-term contamination with certain *L. innocua* and *L. monocytogenes* genotypes.

Some studies indeed allude to wider contamination of dairy facilities. Parisi et al. [36] isolated *Listeria* spp. at 19/34 cheese factories (55.8%). Occasionally, *L. innocua* and *L. monocytogenes* were detected at the same sampling site (2/19 plants) and persisted in floor drains, which were identified as ideal sampling sites to be included in a monitoring system.

Relevantly, we clearly identified a higher fluctuation of *L. innocua* and *L. monocytogenes* genotypes in parallel in 4/5 dairy processing facilities (Figure 1, Table 1). One dairy plant (B) harbored a persistent *L. monocytogenes* genotype (ST121) for seven years without further introduction of other *Listeria* spp. genotypes.

Lomonaco et al. [37] reported two persistent *L. monocytogenes* genotypes in the Gorgonzola processing chain. About 88% of the *L. monocytogenes* strains were serotype 1/2a, which is consistent with our findings (95% of the isolates were serotype 1/2a). However, genotypes were not comparable due to the lack of common nomenclature in *Listeria* spp. subtyping, which is urgently in need of rectification [38]. In this respect, Jagadeesan et al. [39] highlighted the need to include metadata for genotypic approaches, which should be sufficiently cleaned with the removal of replicates and unintended information. Actual studies indicate that whole-genome sequencing (WGS) and core genome (cg) MLST approaches already contribute to the real-time exchange of information on the emergence and geographic dispersal of clones [40,41,42]. Maury et al. [43] reported that *L. monocytogenes* CC1 are strongly associated with dairy products, whereas hypovirulent clones, CC9 and CC121, are related to meat products.

In the presented study, several disease-related genotypes that are globally distributed were introduced into the dairy processing environment (ST1, ST3, ST7, ST59, ST155, ST398, and ST403). However, ST14 and ST121 established themselves for a longer time (7 and 11 years, respectively) in the FPE and tended to be persistent. Almost all of the persistent *L. innocua* and *L. monocytogenes* isolates that we identified were rhamnose-positive (API profiles 7510 and 6510; Table 1). Rhamnose is a naturally occurring monosaccharide present in plant material and important for saprophytes such as *Listeria* spp.

Atypical *L. innocua* and *L. monocytogenes* lacking the ability to ferment rhamnose (where the *pdu* operon for propanediol utilization is missing) are potentially less capable of exploiting nutritional sources important for adaptation to the FPE [44].

Further, atypical strains with deficient rhamnose fermentation have been reported to be attenuated in virulence and have reduced resistance to temperature changes [45]. In contrast, *L. monocytogenes* serotype 1/2a mutants confer phage-resistance due to a loss of rhamnose. This is important when there is a field application of lytic phage cocktails as biocontrol measures [46]. Therefore, detailed characterizations of *L. monocytogenes* and *L. innocua* rhamnose-positive and -negative field strains in respect of persistence in the FPE and resistance to biocontrol measures are important.

Pasquali et al. [47] also identified ST14 and ST121 as persisters in a rabbit meat processing plant. Several strain-specific features, such as a stronger biofilm-forming potential (ST14) and the presence of the *qacH* gene associated with adaptation to BAC in ST121, contributed to successful establishment in the FPE.

The stress survival islet (SSI-1) inserted into the intergenic region *lmo0443* to *lmo0449* in *L. monocytogenes* is present in long-term persister ST14 and is related to acid tolerance [26]. *L. monocytogenes* ST121 and all *L. innocua* isolates that we identified harbored the SSI-2 (2.2 kbp fragment), which is related to elevated tolerance to oxidative and alkaline stress (Table 1) [48].

The entrance and establishment of certain successful genotypes in the FPE are influenced by several factors. These include strain properties (e.g., prophage diversification, transposons, plasmids), the possibility of reintroduced genotypes by raw material, over-diluted biocides on wet surfaces, and a permanent change in cleaning regimes by external cleaning companies and consultants [49]. Consequentially, Muhterem-Uyar et al. (2018) reported that in a heavily contaminated cheese processing environment, the strain variability (ST1, ST7, ST21, and ST37) was reduced to a persistent genotype (ST5) harboring a plasmid type (*plM*80 related), which is present in successful clones worldwide [20].

In corroboration, we observed higher MICs towards biocides in M5[B]=M5[D]=M5[E] (ST121, 1/2a), IN5[A]=IN5[C]=IN5[D]=IN5[E] (ST1597), and IN2[E] (ST637), which were better adapted to benzalkonium chloride (up to two-fold higher). IN1[E] (ST1595) and IN2[E] (ST637) were better adapted to sodium hypochlorite (2.7–5.7-fold higher).

In FPEs, resident *Listeria* are frequently exposed to sublethal concentrations of biocides due to the dilution effect of wet surfaces and the presence of food soil [50]. Therefore, testing the sensitivity of *Listeria* spp. to sublethal concentrations of biocides should be performed routinely to identify potential strain adaptations. Particularly, *L. monocytogenes* and *L. innocua* isolated from the pork processing chain have been shown to harbor efflux pumps and resistance genes *(cadA1-cadA4, arsA1, arsA2)* that confer resistance to benzalkonium chloride and heavy metals [51].

The exchange of genetic material between *L. innocua* and *L. monocytogenes* has been observed in a few studies. The possibility of horizontal gene transfer (HGT) of plasmids, including heavy metal resistance, enhanced tolerance to QACs and DNA intercalating dyes between *L. welshimeri, L. innocua* and *L. monocytogenes,* has been described by sequence analysis in experimental settings and by comparison of FPE isolates [52,53,54].

The presence of atypical hemolytic *L. innocua* strains in the food chain might also have been introduced by HGT, and this constitutes a reservoir of virulence genes transferable to other species [12,55]. This could be the same for genetic exchange between *L. innocua* and *L. monocytogenes* related to environmental adaptation. More research focusing on the uptake of genetic material by *Listeria* spp. in the FPE is warranted.

We identified housekeeping genes common in novel identified *L. innocua* ST1595, ST1597, ST1599, and ST1601 in STs 605 and ST1085 that are recorded in the MLST database, and *L. monocytogenes* genetic lineage III (*abcZ* 25, *bglA* 73, *dapE* 96, *dat* 45) (Table 1, Table S1).

Comparing *L. innocua* STs from this study to the MLST Institute Pasteur database, we identified ST603 (CC600) and ST637 (CC140) (Figure 2), commonly isolated from different niches (e.g., silage, food, human blood samples). Comparing the NCBI available strains (*n* = 11) to *L. innocua* from this study, no complete match was found based on STs. The nearest match was *L. innocua* reference strain CLIP 11,262 assigned to CC140, which differed by the *ldh* housekeeping gene *(ldh* 74) in comparison to ST637 (*ldh* 192) (https://www.ncbi.nlm.nih.gov/genome/genomes/1024?; https://bigsdb.web.pasteur.fr/cgi-bin/bigsdb/bigsdb.pl?db=pubmlst_listeria_isolates).

In fact, the draft genome of *Listeria innocua* UAM003-1A, available from NCBI, is also related to highly abundant CC140 [14].

We did not identify any atypical hemolytic ST188 or ST437 *L. innocua* strains (LIP-1 positive, *hly* positive), which have recently been isolated from wild bird feces in Finland [12] and previously described by Volokhov [56]. The *L. innocua* strains included in our study showed no relationship to the newly announced MEZLIS26 genome, due to their different housekeeping genes. The latter *L. innocua* was assigned to the highly abundant CC537 [15].

*L. innocua* has been reported to be more commonly found in the FPE than *L. monocytogenes* [57], which is supported by our findings. We fully agree with Jemmi and Stephan [58] who suggest that *L. innocua* is a good hygiene indicator and also a marker for unrecognized *L. monocytogenes* contamination events in the FPE. What should now be considered in retrospect is the demanding nature of the microbiological reference method, for example, ISO 11290-1, concerning the differentiation of *L. innocua* and *L. monocytogenes* [59]. Reports focusing on the challenges associated with *Listeria* detection and confirmation are available, including atypical strains or *L. monocytogenes* present in lower concentrations due to competitive *L. innocua* strains during enrichment or that mask detection of *L. monocytogenes* on selective agar plates such as ALOA medium [60,61] (https://www.fda.gov/food/laboratory-methods-food/guidelines-bam-users-identification-atypical-hemolytic-listeria-isolates). This might also have contributed to a higher isolation rate of *L. innocua* compared to *L. monocytogenes* in our study.

In respect of long-term contamination events with *L. monocytogenes* and other *Listeria* species, subtyping methods are helpful tools to identify the true nature of persister candidates.

## 5. Conclusions

*L. monocytogenes* is a foodborne pathogen of significance to human health, and it is able to co-survive in the dairy FPE in microbial communities with other *Listeria* species and with other bacteria (e.g., *Proteobacteria*, lactic acid bacteria) [33,62]. Our study identified for the first time the recurrent isolation and persistence of *L. innocua* in *L. monocytogenes*-colonized habitats. Novel local *L. innocua* sequence types (ST1595 to ST1601) were identified, which shared, to a certain extent, the housekeeping genes that are also common in *L. monocytogenes* genetic lineage III. Either SSI-1 (ST14) or SSI-2 (ST121, all *L. innocua*) were present in strains successfully adapted to the FPE. There is a great need for further insight into the processes of FPE adaptation and exchange of genetic information between *Listeria* species so that appropriate food safety control measures can be designed.

## Figures and Tables

**Table 1:** *L. innocua* (*n* = 139) and *L. monocytogenes* (*n* = 81) isolate characteristics included in this study.

PRODUCER	SOURCE	OCCURRENCE	TIME- FRAME	ISOLATES (*n*)	PFGE PROFILE (*ASC*I/*APA*I)	CC ^f^	ST (Serogroup)	*abcZ*	*bglA*	*cat*	*dapE*	*dat*	*ldh*	*lhkA*	SSI-1 + ^g^	SSI-1- ^h^	SSI-2 ^i^
***L. monocytogenes* (API profile 6-5-1-0)**																	
C	milk filter (PA)	spor^c^	once	1	M9[C]	1 (I)	1 (4b, 4d, 4e)	3	1	1	1	3	1	3	0	1	0
D	smear (PAL)	spor	once	1	M6[D]	3 (I)	3 (1/2b, 3b)	4	4	4	3	2	1	5	1	0	0
A	soft cheese (P)	spor	once	1	M8[A]	7 (II)	7 (1/2a, 3a)	5	8	5	7	6	404	1	1	0	0
E	smear (PAL)	spor	once	1	M4[E]	398 (II)	398 (1/2a, 3a)	7	13	19	6	1	7	1	0	1	0
A	smear (PAL)	spor	once	1	M12[A]	ST529 (III)	529 (4b, 4d, 4e)	25	73	82	96	45	211	67	0	1	0
E	acid curd cheese (P)/smear (PAL)	rec ^b^	2 mo ^d^	2	M1[E]	59 (I)	59 (1/2b, 3b)	11	1	12	16	3	1	7	0	1	0
C	swab (FCS)	rec^c^	1 yr ^d^	2	M10[C]	155 (II)	155 (1/2a, 3a)	7	10	16	7	5	2	1	1	0	0
E	smear (PAL)	rec^c^	7 mo	2	M3[E]	403 (II)	403 (1/2a, 3a)	7	7	10	4	5	24	1	1	0	0
A/D	drain water (EL)/smear (PAL)	rec^c^/spor	11 yr/once	11/1	M11[A]=M7[D]	14 (II)	14 (1/2a, 3a)	8	6	13	6	5	2	1	1	0	0
E	acid curd cheese (P)/culture (PA)/environment (NFCS)	rec ^b^	1 mo	3	M2[E]	121 (II)	121 (1/2a, 3a)	7	6	8	8	6	37	1	0	0	1
B/D/E	smear (PAL)	rec^c^/rec^b^/spor	7 yr/4mo/once	47/7/1	M5[B]=M5[D]=M5[E]	121 (II)	121 (1/2a, 3a)	7	6	8	8	6	37	1	0	0	1
***L. innocua* (API profile 7-5-1-0)**																	
C	smear (PAL)	spor	once	1	IN10[C]	ST1596	1596	26	21	33	33	48	213	216	0	0	1
C	cheese (P)	spor	once	1	IN18[C]	ST530	530	28	62	40	97	45	214	53	0	0	1
A	smear (PAL)	spor	once	1	IN12[A]	140	637	28	23	33	35	23	192	16	0	0	1
C	floor water (NFCS)	spor	once	1	IN15[C]	140	637	28	23	33	35	23	192	16	0	0	1
E	smear (PAL)	rec ^b^	4 mo	2	IN2[E]	140	637	28	23	33	35	23	192	16	0	0	1
E	smear (PAL)	spor	once	1	IN4ST[E]	600	603	36	21	40	108	65	243	81	0	0	1
C/E	hard cheese (P)/acid curd cheese (P)/smear (PAL) environment (NFCS	spor/rec^c^	once/5.6 yr	1/12	IN4[C]=IN4[E] /n. t.^e^	600	603^a^	36	21	40	108	65	243	81	0	0	1
A/C/D/E	grating cheese (P)/smear, brine(PAL)/floor water (EL)	rec ^c^/rec ^c^/rec ^c^/rec ^c^	1 yr/6.8yr/6.2yr/1yr	2/22/68/2	IN5[A]=IN5[C]=IN5[D]=IN5[E]/n. t.	ST1597	1597 ^a^	36	23	30	96	195	19	16	0	0	1
A	smear (PAL)	spor	once	1	IN9[A]	448	448	65	21	40	33	45	170	53	0	0	1
C	raw milk (RM)	spor	once	1	IN14[C]	ST1598	1598	79	21	33	97	20	356	58	0	0	1
A	smear (PAL)	spor	once	1	IN16[A]	ST43	43	143	21	40	167	55	307	16	0	0	1
C	hard cheese (P)	spor	once	1	IN21[C]	ST43	43	143	21	40	167	55	307	16	0	0	1
A/C/D	soft cheese (P)/smear (PAL)	rec ^b^/spor/rec ^b^	5 mo/once/4mo	02.01.2003	IN6[A]=IN6[C]=IN6[D]	ST1599	1599^a^	143	95	30	96	55	180	16	0	0	1
***L. innocua* (API profile 7-1-1-0)**																	
E	acid curd cheese (P)/enrichment (PAL)/drain water (EL)	rec ^c^	6 mo	3	IN1[E]	ST1595	1595 ^a^	25	73	237	130	55	19	16	0	0	1
D	semi-hard cheese (P)	spor	once	1	IN8[D]	ST1482	1482	26	21	40	33	45	19	53	0	0	1
C	cheese (P)	spor	once	1	IN17[C]	ST605	605	36	21	30	35	45	69	17	0	0	1
C	cheese (P)	spor	once	1	IN20[C]	ST605	605	36	21	30	35	45	69	17	0	0	1
C	cheese (P)	spor	once	1	IN19[C]	ST1087	1087	191	21	184	110	45	356	16	0	0	1
C	smear (PAL)	rec ^c^	1.3 yr	2	IN7[C]	ST1085	1085 ^a^	188	157	182	223	136	353	148	0	0	1
E	acid curd cheese (P) smear (PAL)	rec ^c^	6 mo	4	IN3[E]	ST1601	1601 ^a^	250	140	73	223	136	341	214	0	0	1
***L. innocua* (API profile 7-5-3-0)**																	
A	smear (PAL)	spor	once	1	IN13[A]	ST1600	1600	40	62	30	33	55	356	17	0	0	1
A	smear (PAL)	spor	once	1	IN11[A]	ST1008	1008	173	140	173	208	136	341	138	0	0	1

^a^ sporadically isolated *Listeria* spp. genotypes; ^b^ recurrently isolated *Listeria* spp. genotypes; ^c^ recurrently isolated *Listeria* spp. genotypes over a period of ≥6 months defined as persistent; ^d^ mo, month; yr, year; ^e^ non-typable; ^f^ CC, clonal complexes; genetic lineages are provided in brackets; ^g^ SSI-1+, stress survival islet positive strains; ^h^ SSI-1-, stress survival islet negative strains, F2365_0481 homologous gene; ^i^ SSI-2, stress survival islet 2 positive genotypes. Abbreviations: Product associated liquids (PAL; smear, brine), product associated samples (PA, culture, and enrichment), raw material (RM), food contact surfaces (FCS), non-food contact (NFCS) surfaces, and environmental (E) liquid samples (floor and drain water). Red marked housekeeping genes are present in *L. monocytogenes* genetic lineage III and *L. innocua*.

## Supplementary Materials

The following are available online at https://www.mdpi.com/2076-2607/8/2/234/s1.
