# Supplementary material for: Predominance of Distinct Listeria Innocua and Listeria Monocytogenes in Recurrent Contamination Events at Dairy Processing Facilities
Source: Microorganisms. 2020 Feb 10;8(2):234. doi: 10.3390/microorganisms8020234 (PMC7074772; doi:10.3390/microorganisms8020234)
Supplement: Supplementary file 1 [file microorganisms-08-00234-s001.pdf]

Supplement Table S1: ***L.*** *innocua* (*n*=139) and *L. monocytogenes* (*n*=81) isolate characteristics based on multi-locus sequence typing (MLST) included in this study in comparison to the Institut Pasteur MLST isolate database.).

**Housekeeping Gene**	**Species**	**CC (Genetic Lineage)**	**ST (Serogroup)**	**CC**	**Isolates (n)**
**This Study**				**Mlst Database**	
abcZ					
3	*L. monocytogenes*	1 (I)	1 (4b, 4d, 4e)	1, 6, 315, 217, 218, 345, 388	712
4	*L. monocytogenes*	3 (I)	3 (1/2b, 3b)	3	330
11	*L. monocytogenes*	59 (I)	59 (1/2b, 3b)a	59, 77, 195, 224, 426, 489, 517	145
5	*L. monocytogenes*	7 (II)	7 (1/2a, 3a)	7, 8, 26, 37, 177, 204, 229, 321, 380, 415, 570, 645, 739, 787, 906, 912, 1211	643
7	*L. monocytogenes*	121, 155, 398, 403 (II)	121*, 155*, 398, 403* (1/2a, 3a)	11, 14, 18, 19, 21, 31, 33, 89, 101, 121, 155, 193, 207, 307, 375, 398, 403, 451, 475, 573, 842, 940	669
8	*L. monocytogenes*	14 (II)	14* (1/2a, 3a)	14	44
**25**	*L. monocytogenes, L. innocua*	ST529 (III), ST1595	529 (4b, 4d, 4e),1595*	132 (*L. innocua*)	7
26	*L. innocua*	ST1482, ST1596	1482, 1596	133, 602, 1482, 1596	16
28	*L. innocua*	140, ST530	637, 530	140, 537	23
36	*L. innocua*	600, ST605, ST1597	603*, 605, 1597*	492, 600	12
**40**	*L. innocua*	ST1600	1600	267 (*L. monocytogenes*)	3
65	*L. innocua*	448	448	448, 731	4
79	*L. innocua*	ST1598	1598	532	9
143	*L. innocua*	ST43, ST1599	43, 1599*		2
173	*L. innocua*	ST1008	1008		3
188	*L. innocua*	ST1085	1085*		1
191	*L. innocua*	ST1087	1087		1
250	*L. innocua*	ST1601	1601*		2
bglA					
1	*L. monocytogenes*	1, 59 (I)	1, 59 (4b, 4d, 4e)	1, 2, 5, 54, 59, 87, 218, 240, 363, 373, 388, 389, 426, 554, 651	1149
4	*L. monocytogenes*	3 (I)	3 (1/2b, 3b)	3, 392	337

6	*L. monocytogenes*	14, 121 (II)	14*, 121* (1/2a, 3a)	8, 11, 14, 18, 19, 89, 90, 121, 321, 375, 573, 787	536
7	*L. monocytogenes*	403 (II)	403* (1/2a, 3a)	21, 37, 204, 403	114
8	*L. monocytogenes*	7 (II)	7 (1/2a, 3a)	7, 1211	286
10	*L. monocytogenes*	155 (II)	155* (1/2a, 3a)	26, 29, 155, 177, 199, 645, 906	262
13	*L. monocytogenes*	398 (II)	398 (1/2a, 3a)	20, 398, 366	75
21	*L. innocua*	ST43, 448, 600, ST605, ST1087, ST1482, ST1596, ST1598	43, 448, 603*, 605, 1087, 1482, 1596, 1598	133, 492, 532. 537, 600	56
23	*L. innocua*	140, ST1597	637, 1597*	140, 1086	12
62	*L. innocua*	ST530, ST1600	530, 1600	448	4
**73**	*L. monocytogenes*, *L. innocua*	ST529 (III), ST1595	529 (4b, 4d, 4e), 1595*		2
95	*L. innocua*	ST1599	1599*	731	5
140	*L. innocua*	ST1008, ST1601	1008, 1601*		9
157	*L. innocua*	ST1085	1085*		1
			cat		
1	*L. monocytogenes*	1 (I)	1 (4b, 4d, 4e)	1, 217, 218, 240, 688	532
4	*L. monocytogenes*	3 (I)	3 (1/2b, 3b)	3, 87, 195, 373, 379, 517	393
5	*L. monocytogenes*	7 (II)	7 (1/2a, 3a)	7	285
8	*L. monocytogenes*	121 (II)	121* (1/2a, 3a)	26, 121, 229, 307, 321, 369, 412, 475, 570, 573, 645, 787, 842	260
10	*L. monocytogenes*	403 (II)	403* (1/2a, 3a)	11, 31, 124, 207, 403, 912, 940	127
12	*L. monocytogenes*	59 (I)	59 (1/2b, 3b)	4, 54, 59, 88, 224, 284, 344, 388, 389, 392, 489	212
13	*L. monocytogenes*, *L. innocua*	14 (II)	14* (1/2a, 3a)	14, 375	33
16	*L. monocytogenes*	155 (II)	155* (1/2a, 3a)	155	112
19	*L. monocytogenes*	398 (II)	398 (1/2a, 3a)	19, 398	38
30	*L. innocua*	ST605, ST1597, ST1599, ST1600	605, 1597*, 1599*, 1600	132, 133	21
33	*L. innocua*	140, ST1596, ST1598	637, 1596, 1598	140, 731	17
40	*L. innocua*	ST43, 448, ST530, 600, ST1482	43, 448, 530, 603*, 1482	448, 492, 600	18
73	*L. innocua*	ST1601	1601*		3
82	*L. monocytogenes*	ST529 (III)	529 (4b, 4d, 4e)		1

173	*L. innocua*	ST1008	1008		3
182	*L. innocua*	ST1085	1085*		1
184	*L. innocua*	ST1087	1087		1
237	*L. innocua*	ST1595	1595*		2
			dapE		
1	*L. monocytogenes*	1 (I)	1 (4b, 4d, 4e)	1, 554, 651	522
3	*L. monocytogenes*	3 (I)	3 (1/2b, 3b)	3, 4, 5, 6, 217, 315, 345, 379, 392, 426, 489	815
4	*L. monocytogenes*	403 (II)	403* (1/2a, 3a)	9, 204, 307, 403	334
6	*L. monocytogenes*	14, 398 (II)	14*, 398 (1/2a, 3a)	11, 14, 19, 20, 193, 199, 398, 412	275
7	*L. monocytogenes*	7, 155 (II)	7, 155* (1/2a, 3a)	7, 155	429
8	*L. monocytogenes*	121 (II)	121* (1/2a, 3a)	90, 101, 121, 375	234
16	*L. monocytogenes*	59 (I)	59 (1/2b, 3b)	59, 218, 288	84
33	*L. innocua*	448, ST1482, ST1596, ST1600	448, 1482, 1596, 1600	133, 448, 532	24
35	*L. innocua*	140	637	140	11
35	*L. innocua*	ST605	605		
**96**	*L. monocytogenes, L. innocua*	ST529 (III), ST1597, ST1599	529 (4b, 4d, 4e), 1597*, 1599*		3
97	*L. innocua*	ST530, ST1598	530, 1598	537, 1086	18
108	*L. innocua*	600	603*		5
110	*L. innocua*	ST1087	1087		6
130	*L. innocua*	ST1595	1595*	731	4
167	*L. innocua*	ST43	43		1
208	*L. innocua*	ST1008	1008		3
223	*L. innocua*	ST1085, ST1601	1085*, 1601*		2
			dat		
1	*L. monocytogenes*	398 (II)	398 (1/2a, 3a)	11, 19, 398, 412	526
2	*L. monocytogenes*	3 (I)	3 (1/2b, 3b)	2 , 3, 4, 54, 240, 284, 388, 389, 426	839
3	*L. monocytogenes*	1, 59 (I)	1, 59 (4b, 4d, 4e)	1, 5, 6, 59, 77, 87, 88, 195, 217, 344, 345, 379, 392, 489, 506, 517, 651, 1000	921

5	*L. monocytogenes*	14, 155, 403 (II)	14*, 155*, 403* (1/2a, 3a)	8, 14, 20, 21, 89, 155, 204, 403, 739	516
6	*L. monocytogenes*	7, 121 (II)	7, 121* (1/2a, 3a)	7, 26, 90, 121, 124, 177, 321, 380, 475, 573, 906, 1211	622
20	*L. innocua*	ST1598	1598	132, 307, 537	22
23	*L. innocua*	140	637	140	10
**45**	*L. monocytogenes, L. innocua*	ST529 (III), 448, ST530, ST605, ST1087, ST1482	529 (4b, 4d, 4e), 448, 530, 605, 1087, 1482	448, 532, 731, 1086	32
48	*L. innocua*	ST1596	1596	492	4
55	*L. innocua*	ST43, ST1595, ST1599, ST1600	43, 1595*, 1599*, 1600	133	1
65	*L. innocua*	600	603*	600	5
136	*L. innocua*	ST1008, ST1085, ST1601	1008, 1085*, 1601*		5
195	*L. innocua*	ST1597	1597*		2
			ldh		
1	*L. monocytogenes*	1, 3, 59 (I)	1 (4b, 4d, 4e), 3, 59 (1/2b, 3b)	1, 2, 3, 5, 6, 54, 59, 77, 87, 88, 155, 195, 218, 220, 240, 288, 363, 373, 379, 388, 389, 426, 489, 651, 688, 1000	1492
2	*L. monocytogenes*	14, 155 (II)	14*, 155* (1/2a, 3a)	7, 8, 11, 14, 26, 89, 90, 155, 207, 307, 412, 912	495
7	*L. monocytogenes*	398 (II)	398 (1/2a, 3a)	321, 398, 573	88
19	*L. innocua*	ST1482, ST1595, ST1597	1482, 1595*, 1597*	532	6
24	*L. monocytogenes*	403 (II)	403* (1/2a, 3a)	19, 403	24
37	*L. monocytogenes*	121 (II)	121* (1/2a, 3a)	121	162
69	*L. innocua*	ST605	605	133	12
170	*L. innocua*	448	448	448	2
180	*L. innocua*	ST1599	1599*		2
192	*L. innocua*	140	637	140	7
211	*L. monocytogenes*	ST529 (III)	529 (4b, 4d, 4e)		1
213	*L. innocua*	ST1596	1596	140	
214	*L. innocua*	ST530	530		1
243	*L. innocua*	600	603*	600	4
307	*L. innocua*	ST43	43		1

341	*L. innocua*	ST1008, ST1601	1008, 1601*		4
353	*L. innocua*	ST1085	1085*		1
356	*L. innocua*	ST1087, ST1598, ST1600	1087, 1598, 1600		3
404	*L. monocytogenes*	7 (II)	7 (1/2a, 3a)	1211	1
			lhkA		
1	*L. monocytogenes*	7, 14, 121, 155, 403, 398 (II)	7*, 14*, 121*, 155*, 403*, 398 (1/2a, 3a)	7, 8, 9, 11, 14, 18, 19, 20, 21, 26, 29, 31, 33, 89, 121, 124, 155, 177, 193, 199, 204, 207, 229, 262, 307, 380, 398, 403, 412, 451, 475, 570, 573, 645, 906, 912, 940, 1211, 1364	1630
3	*L. monocytogenes*	1 (I)	1 (4b, 4d, 4e)	1, 54, 217, 240, 373	639
5	*L. monocytogenes*	3 (I)	3 (1/2b, 3b)	2, 6, 284, 379, 389, 554	836
7	*L. monocytogenes*	59 (I)	59 (1/2b, 3b)	5, 59	168
16	*L. innocua*	ST43, 140, ST1087, ST1595, ST1597, ST1599	43, 637, 1087, 1595*, 1597*, 1599*	132, 140	27
17	*L. innocua*	ST605, ST1600	605, 1600	133, 532	21
53	*L. innocua*	448, ST530, ST1482	448, 530, 1482	448	4
58	*L. innocua*	ST1598	1598		2
67	*L. monocytogenes*	ST529 (III)	529 (4b, 4d, 4e)		1
81	*L. innocua*	600	603*	600	5
138	*L. innocua*	ST1008	1008		3
148	*L. innocua*	ST1085	108		1
214	*L. innocua*	ST1601	1601*		2
216	*L. innocua*	ST1596	1596		2

**Listeria monocytogenes* and *Listeria innocua* genotypes identified as persistent in cheese producing facilities A-E. Red marked housekeeping genes are present in *L. monocytogenes* genetic lineage III and *L. innocua*. Abbreviations: CC, clonal complex.
